# Universal health coverage saves more lives among severely ill COVID-19 patients: A difference-in-differences analysis of individual patient data in South Korea

**DOI:** 10.1186/s12961-024-01212-9

**Published:** 2024-08-21

**Authors:** Daseul Moon, Jeangeun Jeon, Jieun Park, Min-Hyeok Choi, Myoung-Hee Kim, Hongjo Choi

**Affiliations:** 1 Busan Center for Infectious Disease Control & Prevention, Pusan National University Hospital, Busan, Republic of Korea; 2 Department of Sociology, Yonsei University, Seoul, Republic of Korea; 3 Department of Preventive and Occupational & Environmental Medicine, Pusan National University Medical College, Yangsan, Republic of Korea; 4 Office of Public Healthcare Service, Pusan National University Yangsan Hospital, Yangsan, Republic of Korea; 5 Center for Public Health Data Analytics, National Medical Center, F8, 251 Eulj-Ro, Jung-Gu, Seoul, 04564 Republic of Korea; 6 Division of Health Policy and Management, Korea University College of Health Science, Room 369, B-Dong Hana-Science Building 145 Anam-Ro, Seongbuk-Gu, Seoul, 02841 Republic of Korea

## Abstract

**Background:**

Universal health coverage (UHC) ensures affordability of a variety of essential health services for the general population. Although UHC could mitigate the harmful effects of coronavirus disease 2019 (COVID-19) on patients and their socioeconomic position, the debate on UHC’s scope and ability to improve health outcomes is ongoing. This study aimed to identify the impact of UHC policy withdrawal on the health outcomes of South Korea’s severely ill COVID-19 patients.

**Methods:**

We used a propensity score matching (PSM) and difference-in-differences combined model. This study’s subjects were 44,552 hospitalized COVID-19 patients contributing towards health insurance claims data, COVID-19 notifications and vaccination data extracted from the National Health Information Database and the Korea Disease Control and Prevention Agency from 1 December 2020 to 30 April 2022. After PSM, 2460 patients were included. This study’s exposures were severity of illness and UHC policy change. The primary outcome was the case fatality rate (CFR) for COVID-19, which was defined as death within 30 days of a COVID-19 diagnosis. There were four secondary outcomes, including time interval between diagnosis and hospitalization (days), length of stay (days), total medical expenses (USD) and the time interval between diagnosis and death (days).

**Results:**

After the UHC policy’s withdrawal, the severely ill patients’ CFR increased to 284 per 1000 patients [95% confidence interval (CI) 229.1–338.4], hospitalization days decreased to 9.61 days (95% CI −11.20 to −8.03) and total medical expenses decreased to 5702.73 USD (95% CI −7128.41 to −4202.01) compared with those who were not severely ill.

**Conclusions:**

During the pandemic, UHC may have saved the lives of severely ill COVID-19 patients; therefore, expanding services and financial coverage could be a crucial strategy during public health crises.

**Supplementary Information:**

The online version contains supplementary material available at 10.1186/s12961-024-01212-9.

## Introduction

Universal health coverage (UHC) is crucial for improving population health [1–3], as it ensures financial accessibility of an extensive range of essential health services to the general population. Health coverage expansion in low- and middle-income countries is frequently discussed [4]; however, it is also a critical issue for high-income nations with contribution-based national health insurance (NHI) or fragmented healthcare systems (for example, the USA) [5, 6]. The focus on expansion of health coverage in low-income countries is on improving healthcare access [7, 8], while high-income countries concentrate on its health effects, such as reduction in mortality rates [9, 10].

The experience of the coronavirus disease 2019 (COVID-19) pandemic has raised questions about whether to expand health coverage functions as a crucial health policy during health emergencies. One of the primary discussions revolves around the role of universal health coverage, considered the complete achievement of coverage, in responding to COVID-19 [11–18]. According to UHC advocates, COVID-19 health outcomes improved with the increase of primary care access during the pandemic. However, studies in countries with relatively strong UHC systems, such as Italy, the UK and Spain, are critical of UHC strategies as being insufficient for pandemic response [19, 20]. Empirical studies based on comparative analysis of countries with and without UHC reveal that those with UHC either have lower COVID-19 infection rates or higher case fatality rates (CFR) than countries without UHC systems [11, 21], or no difference is observed [15, 22–24].

However, findings from individual countries, as opposed to cross-country comparative studies, indicate that expanding health coverage played a crucial role in promoting health during the COVID-19 pandemic [16, 25, 26]. An example of this is the COVID-19 treatment subsidy policy in South Korea (herein referred to as Korea). At the onset of the COVID-19 pandemic, South Korea implemented a policy to fully cover the treatment costs for 36 services, including non-reimbursable items, that all COVID-19 patients received during their isolation period. This coverage was provided through the National Health Insurance, national funds and local funds [25, 27]. The policy stipulated that treatment costs would be covered for the entire hospitalization period if patients met the official 20-day isolation release period or showed clinical improvement compared with their pre-COVID-19 condition. Consequently, although limited to the specific disease of COVID-19, this policy represented a realization of universal health coverage (UHC), expanding all three dimensions of coverage: population, services and financial protection [25, 28]. However, as the pandemic prolonged, the Korean government ended the 2-year UHC experiment on 17 December 2021, citing the need for enhancing efficient resource management and reducing government financial burden. While maintaining the policy for severe COVID-19 patients during the isolation period post-diagnosis, the government terminated support after 20 days regardless of clinical improvement, reverting to the pre-existing health insurance system, which covered only 62.6% of medical expenses. Consequently, critically ill patients requiring long-term care incurred additional costs for all medical treatments received after the 20-day period.

To the best of our knowledge, there is limited evidence that has causally identified the effect of UHC on health by analysing individual patient data from severely ill and non-severely ill COVID-19 patients from a high income country with an NHI system. The introduction of South Korea’s COVID-19 medical reimbursement policy and its subsequent withdrawal 2 years later provides a quasi-natural experimental setting to examine the health effects of UHC during a health emergency. For COVID-19 patients, healthcare services became more economically accessible since financial burden due to out-of-pocket costs was removed, specifically, for the severely ill COVID-19 patients who required more prolonged and expensive treatment to recover before the UHC policy withdrawal. This study aimed to determine the impact of UHC policy withdrawal on the fatality rate of severely ill COVID-19 patients using individual patient data.

## Methods

### Data sources and study population

**Fig. 1:**
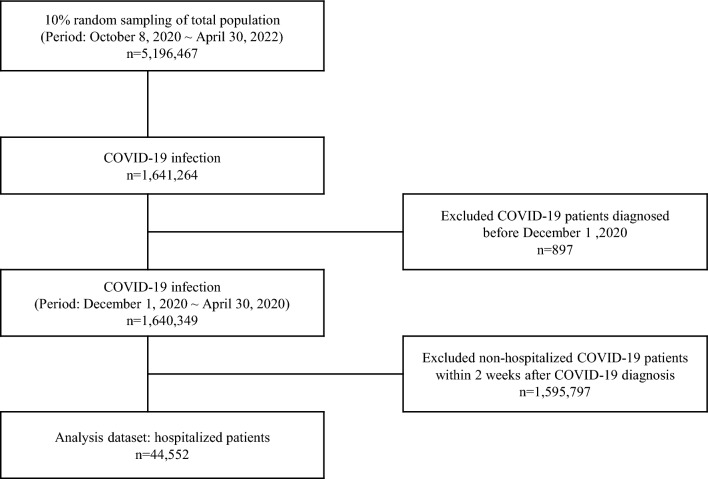
Flow chart of the study population selection including inclusion and exclusion criteria

We combined health insurance claims data, COVID-19 notifications and vaccination data from the National Health Information Database and the Korea Disease Control and Prevention Agency from 1 December 2020 to 30 April 2022 [29]. This study focused on hospitalized patients with COVID-19, most affected by contingent UHC. Through random sampling, 5,196,467 people were selected, representing 10% of the total population on the basis of the date capacity available, and proportionate by sex and age. We identified 1,640,349 individuals with COVID-19 infections. The final population comprised 44,552 patients who were hospitalized within 2 weeks of COVID-19 diagnosis regardless of the main reason of hospitalization (Fig. 1) [30].

### Measures

The primary dependent variable was the fatality rate of COVID-19, defined as death within 30 days of COVID-19 diagnosis. We also analysed the time interval between diagnosis and hospitalization (days), length of stay (days), total medical expenses (USD) and the time interval between diagnosis and death (days). The total medical expenses were calculated by converting 1332.69 Korean Won to 1 US Dollar (USD).

The primary independent variables were group and time. COVID-19 patients were categorized into two groups: (1) hospitalized, severely ill patients (treatment group) and (2) remaining hospitalized patients (control group). The definition of “severely ill” was based on the (WHO grade system defined as higher than ordinal 5, including those with noninvasive mechanical ventilation or a high-flow nasal cannula (grade 5), intubation and invasive mechanical ventilation (grade 6) or invasive mechanical ventilation plus additional support, such as pressors or extracardiac membranous oxygenation (grade 7). The time variable was either the pre-retraction (that is, full UHC, from 1 December 2020 to 16 December 2021) or post-retraction period (that is, usual NHIS coverage, from 17 December 2021 to 30 April 2022).

Other covariates included sex, age, number of vaccinations, disability status, place of residence, household income quintile, Charleston Comorbidity Index, dominant variant [31] and pandemic periods, as these can affect COVID-19-related health outcomes [32].

### Statistical analysis

#### Difference-in-differences with propensity score matching

The causal inferences between UHC and health outcomes were evaluated utilizing difference-in-differences (DID) models with state-level data [33]. However, our study constructed a retrospective individual cohort including the most affected population – severely ill COVID-19 patients – and the least affected population – non-severely affected but hospitalized COVID-19 patients. The setting provided a favourable condition in which to conduct a causal inference between UHC and health outcomes using DID. To reduce potential bias between groups, propensity score matching (PSM) methods were applied with the DID model [34]. Therefore, a quasi-experimental study using PSM and DID models to evaluate the effects of health insurance coverage expansion policies was conducted [35]. Previous review studies have identified potential risk factors of COVID-19 severity, such as age, baseline health conditions [36, 37], socioeconomic position [38] and vaccination [39]. Therefore, we estimated propensity scores using sex, age, number of vaccinations, disability, region, income quintile, Charleston Comorbidity Index (CCI), variant prevalence and pandemic timing as variables that could influence severity. Using the caliper method and the estimated propensity score, the treatment and control groups were matched 1:1 (caliper 0.001). Due to the presence of one cluster in each group, the parallel trend test was not feasible. Therefore, a graphical presentation of the parallel trend of pre-treatment period was done for both groups.

The final estimate was obtained by subtracting the difference in the average CFR between the severely ill and non-severely ill patient populations during the UHC policy period (*t* = 0) from the difference in the average CFR between the severely ill and non-severely ill patients with COVID-19 after moving to the post-UHC policy period (*t* = 1). The estimated regression equation for the DID regression model is presented below:

$${\text{Y}}_{{{\text{it}}}} \, = \,\beta_{0} \, + \,\beta_{{1}} {\text{T}}_{{\text{t}}} \, + \,\beta_{{2}} {\text{I}}_{{\text{i}}} \, + \,\beta_{{3}} {\text{T}}_{{\text{t}}} {\text{I}}_{{\text{i}}} \, + \,\left( {\gamma {\text{X}}} \right)\, + \,{\text{e}},$$

The outcome variable is represented by Y_it_. T_t_ is a time dummy variable that takes the value of 1 after the withdrawal of the UHC policy (after 17 December 2021). I_i_ is a dummy variable indicating whether an individual is severely ill. X is a control variable consisting of PSM variables. β_0_ is the average health outcome of the non-severely ill group before the policy change, β_0_ + β_1_ is the average health outcome of the non-severely ill group after the policy change, β_2_ is the difference between the average health outcomes of the severely ill and non-severely ill groups before the policy change and β_3_ is the value of the double difference test of the average health outcomes of the two groups before and after the policy change. Adjusted DID models included covariates for sex, age, number of vaccinations, disability, region, income quintile, Charleston Comorbidity Index, variant prevalence and pandemic timing. These variables may affect the residuals in the final model even though PSM minimizes their effects. Therefore, a DID analysis with double adjustment for these variables was added to the final model. The Granger plot command in STATA was applied to identify time-specific treatment effects [40].

#### Sensitivity analyses

To avoid the risk of misclassifying major variables, four sensitivity analyses were performed. First, there was a possibility of a mismatch between group allocation and reality because of the upsurge in patients during the Omicron variant-dominant period and after the policy implementation. Some severely ill patients in need of Level 7 care may have received only Level 5 or 6 care. Therefore, a DID test of the CFR was conducted by excluding severely ill Level 7 patients after policy implementation. Second, the policy change may have affected patients who had already reached the 20-day full coverage period at the time of implementation. It is also possible that severely ill patients continued to receive their previous level of financial assistance following the official announcement of the policy until incorporated into the COVID-19 treatment guidelines. Therefore, we further analysed the post-policy change cohort by selecting COVID-19 patients who reached 20 days of hospitalization prior to the policy withdrawal (immediate effect model) and those who were diagnosed after 3 January, 2022, the date of the guideline announcement (delayed effect model). Third, while we defined the CFR as all deaths within 30 days of COVID-19 diagnoses, we also included deaths within 60 and 90 days to account for the possibility of death in severely ill patients after this timeframe. Fourth, the higher CFR for severely ill patients after the policy change may have affected the rapid reduction in length of stay and total healthcare costs. Therefore, we conducted a DID analysis of the length of stay and total healthcare costs, excluding deaths. The statistical analyses were conducted using version 17 of Stata/MP (Stata Corp LLC, College Station, TX, USA). Statistical significance was set at *P* < 0.05.

## Results

The PSM results are presented in Table 1. The treatment and control groups contained 1311 and 43,241 individuals, respectively before matching. After PSM, each group included 1230 patients. As shown in Table 1, covariates between the treatment and control groups, other than disability status had different distributions before PSM matching. After matching, the two groups were comparable in characteristics.

**Table 1:** Characteristics of study population before and after propensity score matching

		Before matching (*N* = 44,552)			After matching (*N* = 2460)		
		Severe (*N* = 1311)	Others (*N* = 43,241)	*P*-value	Severe (*N* = 1230)	Others (*N* = 1230)	*P*-value
		Number (%)	Number (%)		Number (%)	Number (%)	
Age	50 s	320 (24.41)	18,538 (42.87)	< 0.001	314 (25.53)	305 (25.00)	0.975
	60 s	301 (22.96)	7577 (17.52)		280 (22.76)	280 (23.00)	
	70 s	317 (24.18)	7235 (16.73)		298 (24.23)	305 (25.00)	
	80 s	373 (28.45)	9891 (22.87)		338 (27.48)	340 (28.00)	
Sex	Male	734 (55.99)	18,907 (43.72)	< 0.001	680 (55.28)	691 (56.18)	0.655
	Female	577 (44.01)	24,334 (56.28)		550 (44.72)	539 (43.82)	
Vaccination	0	553 (42.18)	8067 (18.66)	< 0.001	481 (39.11)	468 (38.00)	0.672
	1	61 (4.65)	1217 (2.81)		53 (4.31)	47 (4.00)	
	≧ 2	697 (53.17)	33,957 (78.53)		696 (56.59)	715 (58.00)	
Disability	With disability	300 (22.88)	9141 (21.14)	0.128	278 (22.60)	266 (21.63)	0.560
	Without disability	1011 (77.12)	34,100 (78.86)		952 (77.40)	964 (78.37)	
Region	Metropolitan	658 (50.19)	18,027 (41.69)	< 0.001	616 (50.08)	635 (51.62)	0.513
	City	547 (41.72)	20,000 (46.25)		511 (41.54)	506 (41.14)	
	Rural	106 (8.09)	5214 (12.06)		103 (8.37)	89 (7.24)	
Income	1	131 (9.99)	5168 (11.95)	0.030	121 (9.84)	110 (8.94)	0.897
	2	210 (16.02)	7165 (16.57)		195 (15.85)	199 (16.18)	
	3	183 (13.96)	5232 (12.10)		165 (13.41)	161 (13.09)	
	4	179 (13.65)	6691 (15.31)		171 (13.90)	161 (13.09)	
	5	238 (18.15)	7757 (17.94)		221 (17.97)	239 (19.43)	
	6	370 (28.22)	11,300 (26.13)		357 (29.02)	360 (29.27)	
CCI score	0	310 (23.65)	13,204 (30.54)	< 0.001	297 (24.15)	297 (24.15)	0.973
	1	241 (18.38)	8951 (20.70)		230 (18.70)	233 (18.94)	
	2	191 (14.57)	6157 (14.24)		183 (14.88)	181 (14.72)	
	3	161 (12.28)	4600 (10.64)		141 (11.46)	131 (10.65)	
	4	408 (31.12)	10,329 (23.89)		379 (30.81)	388 (31.54)	
Dominant variant	Pre-Delta	169 (12.89)	2542 (5.88)	< 0.001	135 (10.98)	125 (10.16)	0.806
	Delta	529 (40.35)	8645 (19.99)		482 (39.19)	487 (39.59)	
	Omicron	613 (46.76)	32,054 (74.13)		613 (49.84)	618 (50.24)	
Wave	Between waves	425 (32.42)	13,997 (32.37)	< 0.001	394 (32.03)	401 (32.60)	0.132
	Third wave	91 (6.94)	1128 (2.61)		75 (6.10)	61 (4.96)	
	Fourth wave (Delta)	304 (23.19)	4682 (10.83)		270 (21.95)	311 (25.28)	
	Fifth wave (Omicron)	491 (37.45)	23,434 (54.19)		491 (39.92)	457 (37.15)	

**Fig. 2:**
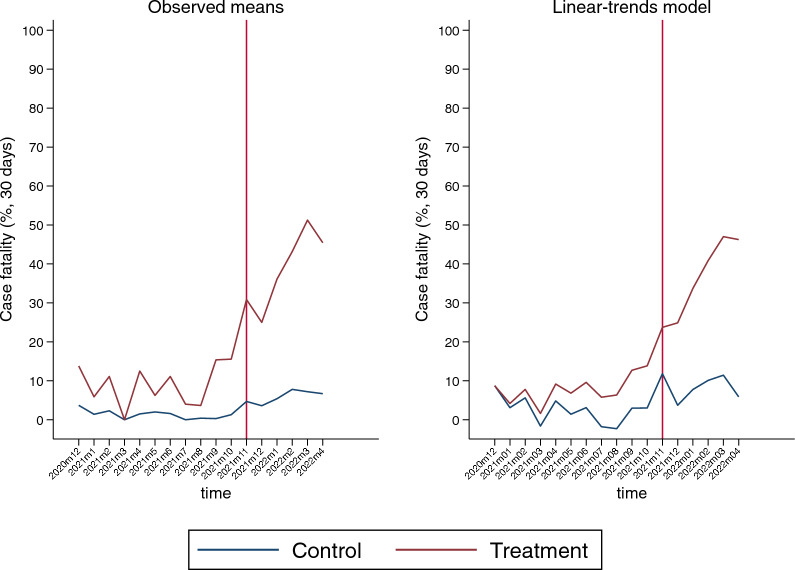
Trends of COVID-19 fatality in treatment and control groups following propensity score matching

Figure 2 depicts the change in CFR in the treatment and control groups during the 17-month study period when PSM was performed. The treatment group had a higher CFR than the control group across all periods. A total of 1 month after the Korean Delta variant dominance period started (August 2021), the CFR in the treatment group increased approximately 4.2 times to 15.38% in September and steadily grew thereafter. The CFR for the control group had been rising since November 2021, but the trend was moderate in comparison to the treatment group.

The distribution of the CFR (percentage) by group characteristics before and after the policy change in the matched sample is shown in Table 2. After the policy was rescinded, the CFR increased significantly, particularly in the treatment group. The CFR in the treatment group increased from 14.47% to 46.28% after the policy was revoked, whereas the CFR for the control group increased from 3.94% to 8.02%.

**Table 2:** Fatality rate in severely ill and other COVID-19 patients before and after policy change (*N* = 2460)

		Before policy change						After policy change					
		Severe (*N* = 532)			Others (*N* = 508)			Severe (*N* = 698)			Others (*N* = 722)		
		Total	Death	*P*-value	Total	Death	*P*-value	Total	Death	*P*-value	Total	Death	*P*-value
		No	Number (%)		No	Number (%)		No	Number (%)		No	Number (%)	
Age	50 s	209	10 (4.78)	< 0.001	204	1 (0.49)	< 0.001	105	17 (16.19)	< 0.001	101	2 (1.98)	0.004
	60 s	140	16 (11.43)		148	6 (4.05)		140	42 (30.00)		132	7 (5.30)	
	70 s	104	18 (17.31)		86	2 (2.33)		194	101 (52.06)		219	14 (6.39)	
	80 s	79	33 (41.77)		70	11 (15.71)		259	163 (62.93)		270	35 (12.96)	
Sex	Male	301	39 (12.96)	0.327	296	11 (3.72)	0.771	379	175 (46.17)	0.972	395	37 (9.37)	0.181
	Female	231	38 (16.45)		212	9 (4.25)		319	148 (46.39)		327	21 (6.42)	
Vaccination	0	172	45 (26.16)	< 0.001	159	14 (8.81)	< 0.001	309	139 (44.98)	0.904	309	42 (13.59)	< 0.001
	1	21	2 (9.52)		23	4 (17.39)		32	14 (43.75)		24	2 (8.33)	
	≧ 2	339	30 (8.85)		326	2 (0.61)		357	170 (47.62)		389	14 (3.60)	
Disability	With disability	69	19 (27.54)	0.006	55	4 (7.27)	0.202	209	98 (46.89)	0.897	211	24 (11.37)	0.052
	Without disability	463	58 (12.53)		453	16 (3.53)		489	225 (46.01)		511	34 (6.65)	
Region	Metropolitan	269	35 (13.01)	0.701	281	13 (4.63)	0.201	347	152 (43.80)	0.518	354	37 (10.45)	0.061
	City	233	37 (15.88)		207	5 (2.42)		278	130 (46.76)		299	15 (5.02)	
	Rural	30	5 (16.67)		20	2 (10.00)		73	41 (56.16)		69	6 (8.70)	
Income	1	42	6 (14.29)	0.630	22	0 (0.00)	0.218	79	44 (55.70)	0.872	88	9 (10.23)	0.897
	2	81	16 (19.75)		77	6 (7.79)		114	50 (43.86)		122	9 (7.38)	
	3	75	13 (17.33)		77	3 (3.90)		90	36 (40.00)		84	8 (9.52)	
	4	82	13 (15.85)		69	2 (2.90)		89	43 (48.31)		92	9 (9.78)	
	5	102	10 (9.80)		112	1 (0.89)		119	52 (43.70)		127	9 (7.09)	
	6	150	19 (12.67)		151	8 (5.30)		207	98 (47.34)		209	14 (6.7)	
CCI score	0	197	17 (8.63)	0.038	188	4 (2.13)	0.002	100	34 (34.00)	0.446	109	10 (9.17)	0.824
	1	119	18 (15.13)		115	1 (0.87)		111	48 (43.24)		118	8 (6.78)	
	2	67	13 (19.40)		64	6 (9.38)		116	62 (53.45)		117	7 (5.98)	
	3	52	6 (11.54)		46	6 (13.04)		89	41 (46.07)		85	6 (7.06)	
	4	97	23 (23.71)		95	3 (3.16)		282	138 (48.94)		293	27 (9.22)	
Dominant variant	pre-Delta	135	15 (11.11)	0.262	125	5 (4.00)	0.968	0	0 (0.00)	0.131	0	0 (0.00)	0.222
	Delta	397	62 (15.62)		383	15 (3.92)		85	29 (34.12)		104	5 (4.81)	
	Omicron	0	0 (0.00)		0	0 (0.00)		613	294 (47.96)		618	53 (8.58)	
Waves	Between waves	248	19 (7.66)	< 0.001	214	3 (1.40)	0.050	146	63 (43.15)	0.215	187	13 (6.95)	0.474
	Third wave	75	9 (12.00)		61	3 (4.92)		0	0 (0.00)		0	0 (0.00)	
	Fourth wave (Delta)	209	49 (23.44)		233	14 (6.01)		61	19 (31.15)		78	4 (5.13)	
	Fifth wage (Omicron)	0	0 (0.00)		0	0 (0.00)		491	241 (49.08)		457	41 (8.97)	

The DID analysis (Table 3) revealed significant differences in the CFR, length of stay and total medical expenses between the two groups before and after UHC policy withdrawal, excluding the time from diagnosis to hospitalization. These differences persisted regardless of covariate adjustment.

**Table 3:** Difference-in-difference test of UHC withdrawal policy impact on COVID-19 fatality and other health-related outcomes (*N* = 2460)

Health outcomes	Crude DID model			Adjusted DID model^a^		
	Coefficient	SE	95% CI	Coefficient	SE	95% CI
30 days fatality (per 1000)	277.00	19.00	(220.00, 334.00)	284.00	28.00	(229.10, 338.40)
Interval between diagnosis and admission (days)	0.12	1.02	(−1.88, 2.12)	0.17	1.02	(−1.84, 2.17)
Duration of admission (days)	−9.72	0.81	−11.31, −8.13)	−9.61	0.81	(−11.20, −8.03)
Total medical expenditure (USD)	−5627.69	735.35	(−7051.36, −4174.61)	−5702.73	727.85	(−7128.41, −4202.01)

^a^Adjusted for age, sex, vaccination, disability, region, income, CCI score, dominant variant and wave

**Fig. 3:**
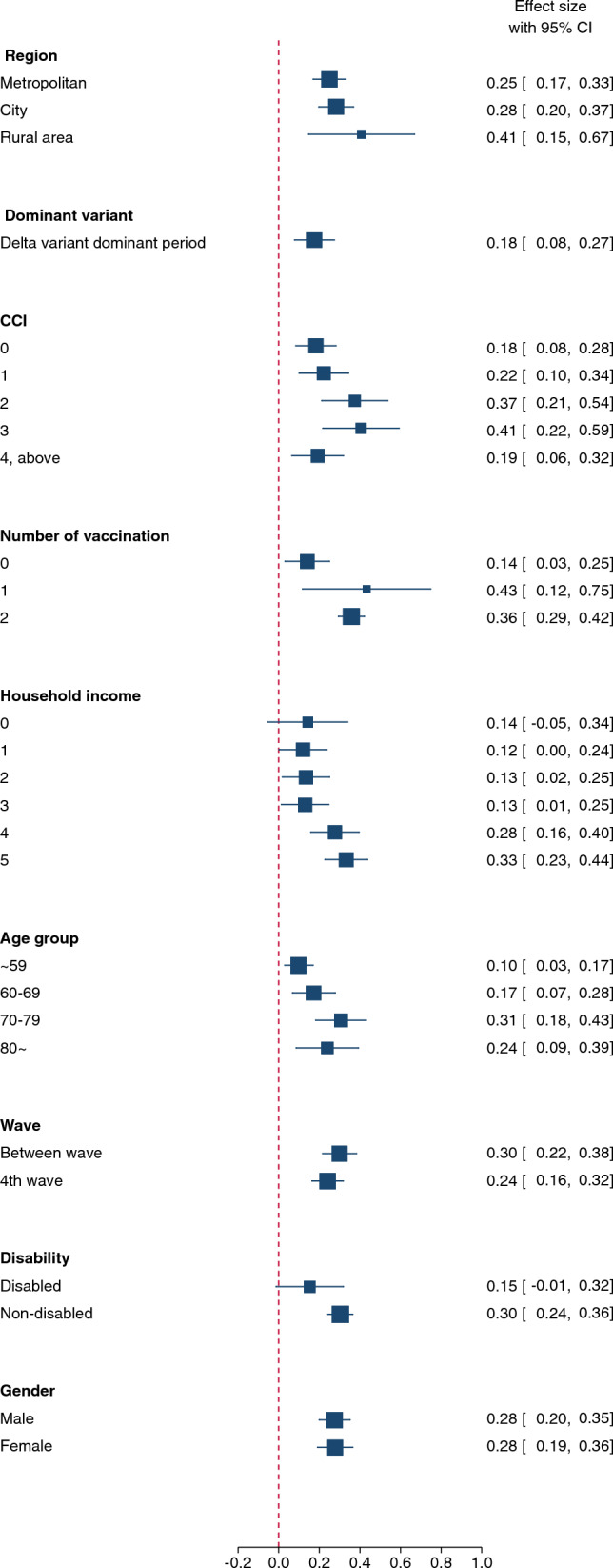
Difference-in-difference analysis of the influence of UHC withdrawal policy on COVID-19 fatalities by subgroups

The subgroup analyses are shown in Fig. 3. After UHC policy withdrawal, the CFR of the treatment group increased relative to that of the control group in all subgroups except for medical aid beneficiaries and people with disabilities.

Sensitivity analyses also demonstrated the differences in CFR, length of stay and total medical expenses between treatment and control groups. An additional file shows results from the analyses in more detail (Additional file 1). It demonstrated that the DID estimate of the CFR per 1000 individuals slightly decreased to 283 (95% CI 228.4–337.7; Table A1) accounting for the time-varying effect of the group variable. Sensitivity analyses by utilizing different timings of the policy change and analysing differences in the length of stay and total medical expenses, excluding deaths, did not demonstrate significant differences from the primary analysis (Tables A2–A4). The differences in CFR between groups within 60 and 90 days after the withdrawal persisted (Table A5).

## Discussion

Our results demonstrate the strategic importance of UHC policies for reducing deaths among severely ill patients during public health emergencies. During the COVID-19 pandemic, the withdrawal of UHC policies led to approximately 284 more deaths per 1000 severely ill COVID-19 patients, indicating that approximately 372 deaths could have been prevented by maintaining UHC for severely ill patients during the observed 17 months.

To the best of our knowledge, our study is the first to causally identify the positive effect of UHC and/or health insurance coverage expansion on health by analysing individual patient data from severely ill and non-severely ill COVID-19 hospitalized patients from a country with an NHI system. Previous studies examining this relationship have been inconsistent, showing differences in disaster-response capacities across countries. Studies have utilized various measures of UHC [11, 15, 21–24, 41]. Additionally, despite general agreement on the definition of UHC, countries have heterogeneous healthcare systems with varying budgets, governance, quality of care, workforce and delivery mechanisms, which may result in inconsistent health outcomes [4, 42]. We overcame these methodological limitations by including a single country to eliminate heterogeneity, measuring actual policy changes and analysing individual-level data.

Our findings are consistent with studies indicating that expanded financial coverage (primary via Medicaid) in the USA during the COVID-19 pandemic improved healthcare access and prevented illness and death [16, 43]. These studies reveal increased out-of-pocket expenditures for COVID-19 hospitalization services among those covered [26, 44]. Another study reported an increase in COVID-19 testing and a decrease in COVID-19-related deaths after the implementation of the UHC policy [45]. However, these studies cannot be used to draw causal inferences about the effects of the policy. Recent research indicates that insurance coverage reduced mortality between waves periods, supporting our findings [9, 10]. A review study concluded that expanding health insurance coverage reduced the financial burden and improved access to care [5], while another review determined that causal inference studies, including randomized controlled trials and DIDs, demonstrate that health-benefit expansion reduces mortality [6].

Third, the withdrawal of the UHC policy decreased healthcare service utilization by severely ill patients, allocating insufficient healthcare resources. These results indicate that full health insurance coverage reduces patients’ financial burden by ensuring access to quality healthcare, thereby preventing deaths [3]. Data on NHI expenditures before and after the withdrawal of the UHC policy revealed an intensive allocation of resources to severely ill COVID-19 patients, who were the primary beneficiaries before the withdrawal and also a significant decrease in expenditures to these patients after withdrawal [28]. The paradoxical finding that hospital mortality was higher among severely ill patients who were not infected with COVID-19 during the pandemic bolsters the importance of resource allocation [46]. These findings are significant because they suggest that a disease-specific approach to UHC may be a risky tradeoff for mortality from other diseases.

Fourth, our subgroup analyses indicate that withdrawing from UHC during a public health emergency escalated amenable mortality for all populations and signifies equity as a core value. We discovered an association between UHC policy withdrawal and increased CFR in all subgroups except medical aid beneficiaries and individuals with disabilities. This suggests that changes in CFR among severely ill COVID-19 patients are mainly attributable to UHC policies rather than socio-environmental factors. Similarly, Lee et al. [25] revealed no differences in COVID-19 CFR during the UHC policy implementation period owing to differences in socioeconomic positions, as Korea’s NHI provides 97% of its population with benefits. Hence, the finding that UHC withdrawal increased CFR across all demographic groups highlights the significance of bolstering services and financial protection with health insurance coverage.

However, the differences in the fatality rate between treatment and control groups due to the withdrawal of UHC were not identified in the populations of medical aid beneficiaries and people with disabilities. This is a significant discovery demonstrating that injustice can still occur under UHC policies. With the withdrawal of the UHC policy, it could be argued that Korea adopted a high-risk strategy that included these groups, thereby preventing deaths among the poor and people with disabilities. Nonetheless, the pre- and post-policy mortality rates were higher in these groups, indicating that pre-existing vulnerabilities continued to function as an exclusionary principle. One study found that COVID-19 excess deaths were higher among (male) medical aid recipients [47], which is consistent with this argument.

Finally, and most importantly, our results used rigorous methodologies to directly refute claims [48, 49] that expanding health insurance coverage does not guarantee amenable mortality reduction. These arguments are frequently based on methodological limitations, such as the power and effective size of studies that correlate the expansion of health insurance coverage and mortality. Our study overcame these limitations by conducting sensitivity analyses as described in the Methodology section. Specifically, we found no changes in results when we analysed our primary analytic variables through different measures.

### Limitations

There are some limitations to our study. First, our analysis did not account for significant healthcare resource factors other than the UHC. For instance, the withdrawal of UHC coincided with an increase in COVID-19 cases, which exacerbated staffing shortages and increased the demand for home care. Consequently, differences in the quality of healthcare before and after withdrawal may have significantly influenced the CFR in our study population. However, due to data limitations, it was difficult to measure potential influencing factors; therefore, we minimized their effects using a DID analysis.

Second, the CFR is the final health outcome, making it difficult to reflect the suffering of severely ill patients from multiple dimensions. For instance, we could not quantify the suffering of caregivers of patients undergoing rehabilitation following prolonged treatment or the suffering of caregivers of severely ill patients from hospitalization to death. This is a significant challenge that requires additional qualitative research, including interviews.

## Conclusions

Our study indicated that UHC may have saved the lives of severely ill COVID-19 patients during the COVID-19-pandemic era. Although specifically limited to COVID-19, South Korea’s contingent UHC program demonstrates that a full three-dimensional health insurance coverage expansion decreases the CFR. Although the policy debate centres on the continuation of universal financial protection, changes in this policy may significantly impact accessibility. Without affordable government support, guardians may hesitate or forgo continuing active treatment for severely ill patients. In a health emergency such as a pandemic, decisions regarding allocating limited resources could directly impact the lives of individuals. Transitioning to an economic mechanism of a price-based demand–supply control to achieve an efficient resource management amid a surge in confirmed COVID-19 cases proved to be a grave problem. Therefore, effective coordination of beds should be accompanied by other actions such as the expansion of primary care, development of triage, securing step-down beds and postponement of elective surgery to care for COVID-19 patients. In this vein, the decision of the South Korean government to withdraw the UHC scheme cost human lives. Future research should explore the role of UHC in improving public health in everyday life, not just during a public health crisis such as COVID-19.

### Supplementary Information

Supplementary material 1. Results from sensitivity analyses.

## Data Availability

The data that support the findings of this study are not available publicly because of the regulation of the National Health Insurance Service (NHIS). Data are available from the Review Board of the National Health Insurance Service (contact via NHIS) for researchers who meet the criteria for access to confidential data. We confirm that none of the authors have any special access or privileges with the NHIS. This study was supported by the Korean government, but anyone who conducts a joint study with a Korean researcher could access NHIS for customized health information data. Applications for data are available through the National Health Insurance Data Sharing website (https://nhiss.nhis.or.kr/bd/ab/bdaba000eng.do), and additional information can be found at a customized health information data webpage (https://nhiss.nhis.or.kr/bd/ab/bdaba032eng.do).
