# Supplementary material for: Universal health coverage saves more lives among severely ill COVID-19 patients: A difference-in-differences analysis of individual patient data in South Korea
Source: Health Res Policy Syst. 2024 Aug 21;22:116. doi: 10.1186/s12961-024-01212-9 (PMC11337885; doi:10.1186/s12961-024-01212-9)
Supplement: Supplementary file 1 — Supplementary material 1. Results from sensitivity analyses. [file 12961_2024_1212_MOESM1_ESM.docx]

**Results from Sensitivity Analyses**

Table A1

Table A2

Table A3

Table A4

Table A5

**Table A1. Sensitivity analysis: DID model of Covid-19 Fatality Excluding level 7 Sick Patients after Policy Change**

|  | | **Crude model** | | | |  | | **Adjusted model** ^a^ | | | |
| --- | --- | --- | --- | --- | --- | --- | --- | --- | --- | --- | --- |
|  |  | **coef.** | | **95% CI** | |  | | **coef.** | | **95% CI** | |
| **Before policy change (A)** | | 105.0 | | (70.5 | 140.2) |  | | 99.0 | | (67.3, | 132.6) |
| **After policy change (B)** | | 383.0 | | (341.5 | 425.0) |  | | 382.0 | | (343.3, | 423.8) |
| B-A | 278.0 | | (220.8 | | 334.9) | |  | | 283.0 | (228.4, | 337.7) |

^a^ Adjusted for age, gender, vaccination, disability, region, income, CCI score, dominant variant, and wave

**Table A2. Sensitivity Analysis: DID Model of Immediate Effect of the Policy Withdrawal**

|  |  | **Crude DID Model** | | |  | **Adjusted DID Model ^a^** | | |
| --- | --- | --- | --- | --- | --- | --- | --- | --- |
|  |  | **Coef.** | **95%CI** | |  | **Coef.** | **95%CI** | |
| **30 days fatality (/1,000)** |  | 257.0 | (196.6, | 316.9) |  | 267.0 | (209.7, | 324.5) |
| **Interval between diagnosis and admission (Days)** |  | 0.80 | (-1.30, | 2.90) |  | 0.81 | (-1.29, | 2.90) |
| **Duration of admission (Days)** |  | -9.70 | (-11.36, | -8.03) |  | -9.59 | (-11.24, | -7.93) |
| **Total medical expenditure (USD)** |  | -6,378.05 | (-7,878.77, | -4,890.01) |  | -6,378.05 | (-7,878.77, | 4,901.34) |

^a^ Adjusted for age, gender, vaccination, disability, region, income, CCI score, dominant variant, and wave

**Table A3. Sensitivity Analysis: DID Model of Delayed Effect of the Policy Withdrawal**

|  | **Crude DID Model** | | |  | **Adjusted DID Model ^a^** | | |
| --- | --- | --- | --- | --- | --- | --- | --- |
|  | **Coef.** | **95%CI** | |  | **Coef.** | **95%CI** | |
| **30 days fatality (/1,000)** | 276.0 | (219.8, | 332.5) |  | 275.2 | (221.2, | 329.2) |
| **Interval between diagnosis and admission (Days)** | -0.29 | (-2.27, | 1.68) |  | -0.22 | (-2.19, | 1.76) |
| **Duration of admission (Days)** | -9.67 | (-11.24, | -8.10) |  | -9.65 | (-11.21, | -8.09) |
| **Total medical expenditure (USD)** | -5,327.55 | (-6,761.36, | -3,914.13) |  | -5,354.97 | (-6,773.15, | -3,936.78) |

^a^ Adjusted for age, gender, vaccination, disability, region, income, CCI score, dominant variant, and wave

**Table A4. Sensitivity Analysis: DID Model after Excluding for Deaths**

|  | | **Crude DID Model** | | |  | **Adjusted DID Model** | | |
| --- | --- | --- | --- | --- | --- | --- | --- | --- |
|  |  | **Coef.** | **95% CI** | |  | **Coef.** | **95% CI** | |
| **Duration of admission (Days)** | **Before policy change** | 8.28 | (7.10, | 9.46) |  | 8.28 | (7.13, | 9.44) |
|  | **After policy change** | 0.89 | (-0.51, | 2.30) |  | 1.4 | (0.03, | 2.78) |
|  | **Difference in differences** | -7.39 | (-9.23, | -5.54) |  | -7.15 | (-8.96, | -5.34) |
| **Total medical expenditure (USD)** | **Before policy change** | 10,505.03 | (9,229.42, | 12,455.96) |  | 10,880.21 | (9,304.45, | 12,455.96) |
|  | **After policy change** | 5,477.62 | (4,837.98, | 6,136.78) |  | 5,529.52 | (4,872.79, | 6,186.25) |
|  | **Difference in differences** | -5,327.55 | (-7,060.03, | -3,669.14) |  | -5,543.71 | (-7,243.64, | -3,843.77) |

^a^ Adjusted for age, gender, vaccination, disability, region, income, CCI score, dominant variant, and wave

**Table A5. DID Test of UHC Withdrawal Policy Impact on 60 Days And 90 Days Covid-19 Case Fatality**

|  | **Before** | | |  | **After** | | |  | **Difference in Differences** | | |
| --- | --- | --- | --- | --- | --- | --- | --- | --- | --- | --- | --- |
|  | **Difference: Treated-Control** | | |  | **Difference: Treated-Control** | | |  |  |  |  |
|  | **Coef.** | **95%CI** | |  | **Coef.** | **95%CI** | |  | **Coef.** | **95%CI** | |
| **60 days case fatality (/1,000)** | 169 | 128.1 | 209.4 |  | 434 | 390.4 | 477.1 |  | 265 | 203.5 | 326.5 |
| **90 days case fatality (/1,000)** | 189 | 147.4 | 231.3 |  | 432 | 387.4 | 475.7 |  | 242 | 179.4 | 305.1 |
